# Circulating bone morphogenetic protein-9 in relation to metabolic syndrome and insulin resistance

**DOI:** 10.1038/s41598-017-17807-y

**Published:** 2017-12-13

**Authors:** Xiaohui Xu, Xiaoqiang Li, Gangyi Yang, Ling Li, Wenjing Hu, Lili Zhang, Hua Liu, Hongting Zheng, Minghong Tan, Danping Zhu

**Affiliations:** 1 Department of Endocrinology, Chongqing Traditional Chinese Medicine Hospital, Chongqing, China; 2 Department of Clinical Laboratory, Children’s Hospital of Chongqing Medical University, Chongqing, China; 3 Department of Endocrinology, the Second Affiliated Hospital Chongqing Medical University, Chongqing, China; 4 Key Laboratory of Diagnostic Medicine (Ministry of Education) and Department of Clinical Biochemistry, College of Laboratory Medicine, Chongqing Medical University, Chongqing, China; 5 Chongqing Prevention and Treatment Hospital for Occupational Diseases, Chongqing, China; 6 Department of Pediatrics, University of Mississippi Medical Center, 2500 North State Street, Jackson, Mississippi, MS 39216-4505 USA; 7 Department of Endocrinology, Xinqiao Hospital, Third Military Medical University, Chongqing, China

## Abstract

Our objective is to determine circulating Bone morphogenetic protein-9(BMP-9) levels in subjects with Metabolic Syndrome (MetS) and examine the relationship between BMP-9 and conventional markers for MetS and insulin resistance (IR). A total of 362 newly diagnosed patients with MetS along with healthy controls were recruited for this cross-sectional study. Circulating BMP-9 levels were measured by ELISA. Circulating BMP-9 levels were significantly lower in MetS patients compared to those of the healthy controls. BMP-9 was associated negatively with Waist hip ratio (WHR), fasting blood glucose (FBG), 2-hour blood glucose after glucose overload (2h-OGTT), HbA1c, triglyceride (TG) levels and HOMA-IR and positively with free fatty acid (FFA) and HDL after control for age and sex. In a multiple linear regression, BMP-9 was independently associated with type 2 diabetes mellitus (T2DM), HOMA-IR and FFA. Binary logistic regression showed that plasma BMP-9 concentrations were significantly associated with MetS even after controlling for anthropometric variables and lipid profiles. In addition, circulating BMP-9 levels reduced progressively with an increasing number of MetS components. The best cutoff values for circulating BMP-9 to predict MetS was 56.6 ng/L. Circulating BMP-9 levels were associated with the key components of MetS and IR.

## Introduction

Metabolic Syndrome (MetS) is characterized by a cluster of risk factors for insulin resistance (IR) type 2 diabetes mellitus (T2DM) and cardiovascular disease, including obesity, hypertension, hyperglycemia, and dyslipidemia^1^. Individuals with MetS have a 24-fold increased risk for the development of T2DM^2^ and are known to be more susceptible to the development of cardiac vascular diseases^3^ and cancers^4,5^. Due to the increasing prevalence of MetS, especially in developing countries^6–8^, it is urgent to discover new strategies for the assessment and treatment of MetS. However, while insulin resistance (IR) is considered to be a major factor in MetS, the exact pathophysiology is unknown although complex genetic, metabolic and environmental factors do play a role^9^. Therefore, it is important to study all factors related to MetS in depth.

Bone morphogenetic protein-9 (BMP-9), a member of transforming growth factor (TGF-β) superfamily, was initially found in hepatocytes and intrahepatic biliary epithelial cells^10^. Subsequently, an additional pattern of distribution was reported^11,12^. BMP-9 was originally identified as relating to chondrogenic and osteogenic factors^13^. Recently, however, some animal studies have demonstrated that BMP-9 has multiple functions including angiogenesis, promoting the differentiation of cholinergic neurons in the central nervous system (CNS) and regulating hepatic growth^14–16^. BMP-9 has also been shown to regulate the key enzymes of fatty acid synthesis in the liver, promote insulin release from the pancreas, suppress hepatic glucose production (HGP) and increase brown adipogenesis in adipose tissue^17,18^. In our recently published study, we showed that circulating BMP-9 levels are significantly lower in newly diagnosed patients with T2DM (nT2DM) than in healthy subjects and BMP-9 levels are associated with HbA_1_c_,_ fasting blood glucose (FBG), 2-h plasma glucose after glucose overload (2-h OGTT, the area under the curve for glucose (AUC_glucose_) and homeostasis model assessment of insulin resistance (HOMA-IR)^19^. However, to date, there have been no studies that accurately assess the clinical relevance of the relationship between BMP-9 and MetS.

In the current study, our aim is to assess the association between circulating BMP-9 levels and MetS as well as the number of components and plasma BMP-9 levels in middle-aged and older Chinese populations.

## Results

### Anthropometric and metabolic parameters in study subjects

A total of 362 participants were enrolled for the analysis. Baseline characteristics of the study subjects are shown in Table 1. No significant differences were observed in age and sex between control subjects and MetS patients. Compared with controls, Mets subjects had higher BMI, FAT (%), Waist hip ratio (WHR), Systolic blood pressure (SBP), Diastolic blood pressure (DBP), triglyceride (TG), cholesterol (TC), Low-density lipoprotein cholesterol (LDL-C), free fatty acid (FFA), HBA1c(%), FBG, 2-h post-glucose load blood glucose (2h-PBG), FIns, HOMA-IR and lower High-density lipoprotein cholesterol (HDL-C) (*P* < 0.01 or *P* < 0.05). After control for age and sex, the differences still existed.

**Table 1:** Main clinical features in MetS and control subjects.

Variable	Controls (n = 147)	MetS (n = 215)	P
Age (yr)	53.8 ± 9.2	54.6 ± 8.2	0.124
Sex (femal %)	59.9% (88, 59)	54.4% (117, 98)	0.332
BMI (kg/m^2^)	22.9 ± 3.0	25.4 ± 2.6	<0.001
FAT (%)	26.3 ± 5.9	31.3 ± 7.0	<0.001
WHR	0.87 ± 0.08	0.93 ± 0.06	<0.001
SBP (mmHg)	116.8 ± 15.0	131.8 ± 18.6	<0.001
DBP (mmHg)	73.5 ± 9.6	82.6 ± 12.1	<0.001
TC (mmol/L)	4.59 ± 1.03	4.90 ± 1.35	0.021
TG (mmol/L)	1.15 (0.80–1.50)	2.00 (1.40–2.70)	<0.001
HDL-C (mmol/L)	1.40 ± 0.60	1.10 ± 0.25	<0.001
LDL-C (mmol/L)	2.67 ± 0.76	2.90 ± 0.98	0.015
FFA (µmol/L)	0.51 (0.38–0.65)	0.64 (0.44–0.90)	<0.001
HBA1c (%)	5.6 ± 0.4	7.1 ± 1.9	<0.001
FBG (mmol/L)	5.2 ± 0.6	7.8 ± 4.0	<0.001
2h-BG (mmol/L)	6.3 ± 1.2	13.2 ± 8.2	<0.001
FIns (mU/L)	8.6 (5.6–13.0)(143)	15.8 (9.5–22.7)(174)	<0.001
HOMA-IR	1.97 (1.30–3.02)(143)	5.04 (3.66–6.75)(174)	<0.001

BMI, Body mass index; FAT (%), the percentage of fat *in vivo*; WHR, Waist hip ratio; SBP, Systolic blood pressure; DBP, Diastolic blood pressure; TG, Triglyceride; TC, Total cholesterol; HDL-C, High- density lipoprotein cholesterol; LDL-C, Low-density lipoprotein cholesterol; FFA, free fatty acid; HbA1c, Glycosylated hemoglobin; FBG, Fasting blood glucose; 2h-BG, 2 h post-glucose load blood glucose; FIns, Fasting plasma insulin; HOMA-IR, HOMA-insulin resistance index. Values are means ± SD or median (interquartile Range).

### Subgroup analysis

**Figure 1:**
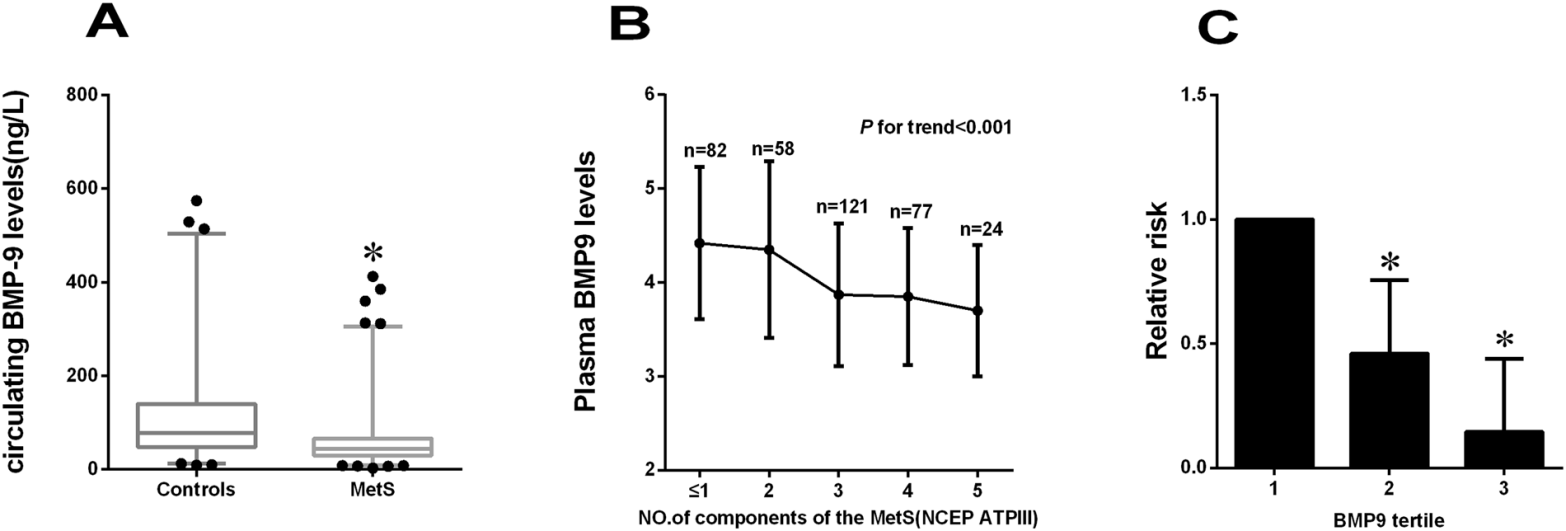
(**A**) Circulating BMP-9 levels in control and MetS individuals. (**B**) Circulating BMP-9 levels in relation to the number of MetS components. The values of BMP-9 are log-transformed. (**C**) Prevalence of elevated MetS in different quartiles f BMP-9: tertile 1, ≤ 39.49 ng/L; tertile 2, 39.49–75.93 ng/L; tertile 3, > 75.93 ng/L (*P < 0.01 *vs*. controls or tertile 1).

In the analysis of subgroup, fasting BMP-9 levels were similar in both male and female (Table 2), suggesting that this hormone does not exhibit distinct sexual dimorphism. Subjects with central obesity, defined by the MetS (NCEP ATPIII) Asian criteria, had significantly lower circulating BMP-9 levels than lean individuals in the study population (*P* < 0.05, Table 2). In addition, individuals with dyslipidemia or hypertension had significantly lower plasma BMP-9 levels than those without (all *P* < 0.01, Table 2). Importantly, fasting circulating levels of BMP-9 were significantly lower in MetS subjects compared with healthy controls [44.31(29.77, 66.39) *vs*. (78.39 (47.78, 140.29) ng/L, *P* < 0.01; Table 2, Fig. 1A]. To further explore the relationship between BMP-9 and the MetS, we stratified the mean levels of circulating BMP-9 by the number of components of the MetS. We found that circulating concentrations of BMP-9 significantly decreased with more than 3 components of MetS (*P* for trend < 0.01, Fig. 1B). Subjects with more components of MetS had decreased BMP-9 levels (BMP-9 levels were log transformed, mean ± SD) of 4.41 ± 0.81; 4.36 ± 0.94; 3.87 ± 0.76; 3.85 ± 0.73; 3.70 ± 4.36, respectively.

**Table 2:** Plasma BMP-9 levels according to gender and various clinical phenotypes.

Clinical phenotype	n	BMP-9 (ng/L)	*P*-value
Female	205	54.75(34.76,88.38)	
Male	157	51.38(31.20,97.13)	0.789
Central obesity	237	50.93(32.01,83.48)	
Non-central obesity	125	61.58(38.71,102.31)	<0.05
Dyslipidemia	270	49.73(32.11,84.29)	
Normal lipid	92	75.72(45.93,109.00)	<0.001
IR	143	49.17(32.27,82.57)	
Non-IR	174	68.48(43.94,114.51)	<0.001
Hypertension	193	49.72(31.20,81.25)	
Normal BP	169	59.75(35.19,110.75)	<0.001
MetS	215	44.31(29.77,66.39)	
Non-MetS	147	78.39(47.78,140.29)	<0.001

IR, insulin resistance; BP, blood pressure. Data are expressed as median (interquartile range).

### Circulating BMP-9 level and its association with anthropometric and biochemical parameters in study subjects

Next, we further analyzed the correlations of plasma BMP-9 levels with anthropometric and metabolic parameters by using spearman correlations. We found that circulating BMP-9 levels negatively correlated with marker of adiposity (WHR), IR marker (HOMA-IR), parameters of glucose metabolism (FBG, 2h-PBG and HbA1c) and parameters of blood fat (TG), while positively correlated with HDL-C and FFA (*P < *0.05 or *P* < 0.01, Table 3). All these correlations remained statistically significant after further adjustment for age and sex in partial correlations. There were no correlations between circulating BMP-9 with fasting and 2 h post-glucose load insulin levels. Multiple stepwise regression analysis showed that FFA, T2DM and HOMA-IR were independent related factors with plasma BMP-9 levels (Table 4). The multiple regression equation was: *Y*_BMP-9_ = 4.469 + 0.160 *X*_FFA_ − 0.294*X*_T2DM_ − 0.296*X*_HOMA-IR_ (*P* < 0.01, R^2^ = 0.10).

**Table 3:** The correlation analysis of plasma BMP-9 levels with other variables in study subjects.

Variable	*r*	*P*
Age (yr)	−0.082	0.125
BMI (kg/m^2^)	−0.047	0.378
WHR	−0.145	<0.001
FAT (%)	−0.099	0.062
SBP (mmHg)	−0.017	0.745
DBP (mmHg)	−0.024	0.649
FBG (mmol/L)	−0.154	<0.001
2h-OGTT (mmol/L)	−0.232	<0.001
HbA1c (%)	−0.161	<0.001
TG (mmol/L)^a^	−0.116	0.031
TC (mmol/L)	0.016	0.767
HDL-C (mmol/L)^a^	0.188	<0.001
LDL-C (mmol/L)	0.034	0.538
FFA (µmol/L)	0.189	<0.001
HOMA-IR^a^	−0.150	<0.001
FIns (mU/L)	−0.024	0.666

In pearson correlation analysis, values included for analysis were age, BMI, WHR, BP, FBG, insulin, HOMA-IR, FFA, total cholesterol, HDL-C, LDL-C, triglyceride. ^a^log-transformed values.

**Table 4:** Multiple stepwise regression showing variables with significant independent associations with plasma BMP-9.

	Coefficient β	95%CI	*P*
Age	−0.013		0.826
Sex	0.143		0.886
FFA	0.160	0.029–0.291	<0.01
Central obesity	−0.020		0.853
Dyslipidemia	−0.049		0.842
T2DM	−0.294	−0.497–(−0.090)	<0.01
HOMA-IR	−0.296	−0.496–(−0.105)	<0.01

BMP-9 was log-transformed before analysis.

### The predictive value of circulating BMP-9 in detecting MetS and dyslipidemia

**Figure 2:**
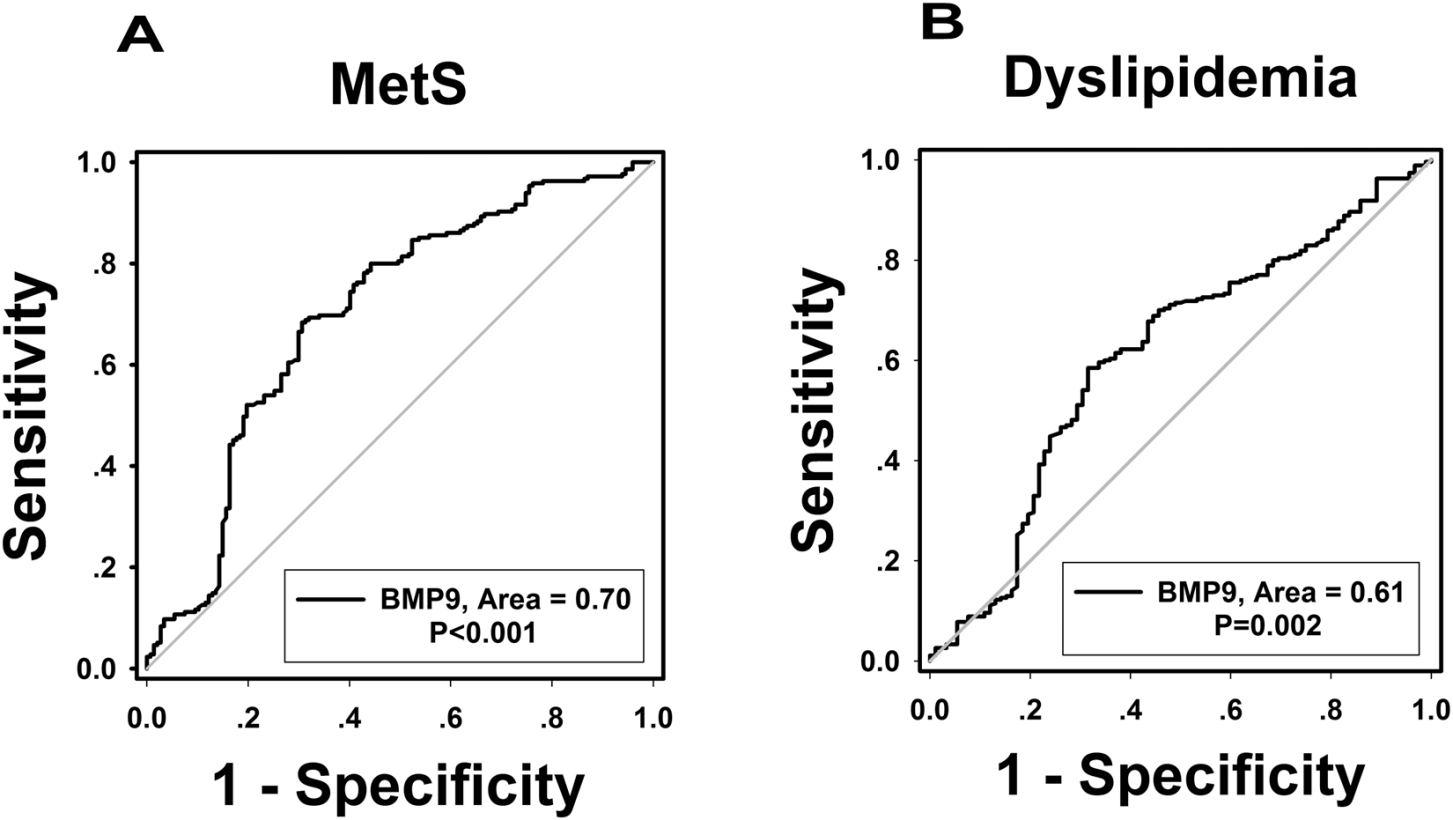
ROC curve analysis was performed for the prediction of MetS **(A)** and dyslipidemia **(B)**.

Binary logistic regression showed that plasma BMP-9 concentrations were significantly associated with MetS even after controlling for anthropometric variables and lipid profile (Table 5). In addition, we divided BMP-9 into three tertiles according to the BMP-9 concentration of the study population (tertile 1, ≤39.49 ng/L; tertile 2, 39.49–75.93 ng/L; tertile 3, >75.93 ng/L) and odds of developing MetS were calculated using logistic regression analysis. When BMP-9 levels were in the tertile 2 and tertile 3, the odds ratios of developing MetS were 0.461 (95% CI 0.259; 0.819) and 0.145 (95% CI 0.081; 0.258), respectively (*vs*. tertile 1, both *P* < 0.01; Fig. 1C). When concentrations were analyzed by row mean score difference and the Cochran-Armitage trend test (Table 6), decreasing BMP9 levels showed a significant linear trend and were independently associated with MetS. Finally, we performed the ROC curve of BMP-9 circulating levels in predicting MetS and dyslipidemia (Fig. 2). The area under the ROC curves was 0.70 (*P* < 0.001) with a sensitivity of 69.4%, specificity of 68.4% for MetS (Figs. 2A) and 0.61 (P < 0.01) with a sensitivity of 68.5%, specificity of 58.5% for dyslipidemia (Fig. 2B). The best cutoff values for circulating BMP-9 to predict MetS and dyslipidemia were 56.6 and 54.9 ng/L.

**Table 5:** Association of circulating BMP-9 levels with MetS in fully adjusted models.

Model adjust	MetS			
	OR	95%CI		*P*
Age, Sex	0.529	0.393–0.711	<0.001	
Age, Sex, BMI, WHR, FAT (%)	0.497	0.3543–0.699	<0.001	
Age, Sex, BMI, WHR, FAT (%), SBP, DBP	0.432	0.295–0.632	<0.001	
Age, Sex, BP, BMI, WHR, FAT(%), lipid profile	0.361	0.224–0.582	<0.001	

Results of binary logistic regression analysis are presented as the odds ratio (OR) of being in MetS status decrease in circulating. BMI, body mass index; WHR, waist-to-hip; FAT (%), the percentage of fat *in vivo*; SBP, systolic blood pressure; DBP, diastolic blood pressure; lipid profile, including total cholesterol, FFA, triglyceride, LDL- and HDL-cholesterol.

**Table 6:** Row mean scores and Cochran–Armitage trend test of the impact of plasma BMP-9 level on MetS

Model adjusted	MetS	
	χ^2^	*P*-value
Row Mean Scores Test	12.2353	<0.001
Cochran-Armitage Trend Test	12.1915	<0.001

The circulating BMP-9 levels of all subjects were cut-off and adjusted for age, sex, BMI, WHR, SBP, DBP, TC, TG, LDL-C and HDL-C.

## Discussion

The major findings of the present study are 1) circulating BMP-9 levels are lower in subjects with MetS compared with healthy controls; 2) circulating concentrations of BMP-9 significantly decreased with more than 3 components of MetS; 3) plasma BMP-9 concentrations are significantly associated with MetS even after controlling for anthropometric variables, lipid profiles and hormone levels.

This study is the first report to analyze circulating BMP-9 levels with ELISA method in MetS subjects. Here, we showed that circulating BMP-9 levels in MetS are significantly decreased compared with the healthy controls, which is similar to a recent study of T2DM patients^19^. In a subgroup analyses, we found that central obesity, dyslipidemia, hypertension and IR subjects also had lower circulating BMP-9 levels compared with controls. These findings further suggest that plasma BMP-9 might be relative to adiposity and obesity-related metabolic diseases, such as MetS.

BMP-9 has been shown to be a regulator of glucose and lipid metabolism^20^. *In vitro*, BMP-9 inhibited the expression of phosphoenolpyruvate carboxykinase (PEPCK), increased fatty acid synthase (FAS) and activated the serine/threonine kinase AKT. In normal and diabetic mice, BMP-9 was shown to decrease glycemia^17^. Furthermore, treatment with an anti-BMP-9 antibody in fasting rats induced glucose intolerance and IR^20^. These data suggest a beneficial effect of BMP-9 on glucose and lipid metabolism and IR. Therefore, the reduction of circulating BMP-9 in subjects with MetS might be due to the increasing consumption in human body to counteract the metabolic stress imposed by MetS, including hyperinsulinemia, hyperglycemia and dyslipidemia. In addition, the decreased levels of BMP-9 may also be a result of both decreased formation and increased degradation in an IR state. However, the cross-sectional nature of the current study does not permit us to infer the causal relationship between BMP-9 and MetS. Therefore, a follow up study will be necessary.

Dyslipidemia and hyperglycemia have been shown to be two important components of MetS and have been added to the MetS definition. This is because it has been suggested that the disease progression in MetS subjects towards T2DM and cardiovascular diseases (CVD) is accompanied by increased metabolic abnormalities. Our data demonstrates that circulating BMP-9 levels in MetS patients are significantly correlated with FBG, 2h-PBG, HbA1c, FFA, HDL and TG, even after adjustment for age and sex. These results also suggest that prolonged hyperglycemia and hyperlipidemia in MetS patients are associated with decreased circulating BMP-9 levels. The positive correlation of BMP9 levels with HDL and negative correlation with HOMA-IR are concordant with the relationship between nonalcoholic fatty liver disease (NAFLD) and those same parameters^21^. Therefore, we speculate that it is possible that low levels of BMP-9 predict NAFLD. Our multiple stepwise regression analysis has identified the FFA, T2DM and HOMA-IR, as significant independent contributors to circulating BMP-9 levels. This clinical evidence suggests that BMP-9 may play a regulatory role in glucose and lipid metabolism in humans, in keeping with the findings of an animal study that suggested a role of BMP-9 in the regulation of glucose and lipid metabolism^18^. Taken together, these findings raise the possibility that BMP-9 acts as a mediator that links glucose and lipid metabolism, IR and MetS. Although it would be premature to conclude a causal effect of BMP-9 on these parameters, it would be of interest to explore whether interventions that specifically raise circulating BMP-9 levels would ameliorate metabolic disorder in MetS subjects.

MetS is a collection of abnormalities that increases the occurrence of CVD and T2DM. To investigate how BMP-9 is associated with CVD and T2DM risk, we compared the relationship between circulating BMP-9 levels and MetS components. We found that circulating concentrations of BMP-9 decreased progressively with continued increases in the number of MetS components. In particular, circulating BMP-9 levels positively correlated with LDL-C, an important risk factor for CVD. This suggests that low BMP-9 levels in MetS subjects may increase cardiovascular risk. In our ROC curve analysis, the results show that circulating BMP-9 may predict both MetS and dyslipidemia in our study population. However, the range of AUC (0.6–0.7) was considered to be of mild-to-moderate significance. Some possible explanation for this relatively weak result, include the sample size and a non-normal distribution of BMP-9 levels in study population. In addition, it is also possible that circulating BMP-9 may not be a good marker for predicting MetS. Therefore, further extensive studies are necessary.

Our study has several limitations. First, this cross-sectional study could not directly determine a causal relationship between BMP-9 and MetS. However, the crossover design minimizes inter-individual differences and ensures that the observed effects of BMP-9 are not attributable to differences in the study population; Second, the sample size was relatively small. However, it provided more than an 80% power to detect significant associations at the *r* > 0.20 and the conventional *P* = 0.05 level; Third, this study is confined to specific age groups and a single race. The homogeny of our population sample decreases the generalizability of our results and decreases the applicability of the associations observed here between BMP-9 and MetS across populations. Therefore, future studies with larger sample sizes examining BMP-9 in various conditions and populations will be needed.

In summary, the results of the current study demonstrate that circulating levels of BMP-9 are decreased in subjects with MetS and associated with measures of IR (HOMA-IR), marker of adiposity (WHR), parameters of glucose metabolism (FBG, 2h-PBG and HbA1c) and parameters of blood fat (TG, HDL and FFA), and the presence of MetS. The strong negative associations of circulating BMP-9 with key components of MetS suggest that BMP-9 may be a potential prediction for risk assessment for cardiovascular disease in obese subjects.

## Materials and Methods

### Subjects

This cross-sectional study was conducted from May 2015 to December 2016. A total of 362 subjects with newly diagnosed MetS patients (nMetS group, n = 215) and healthy controls (NGT group, n = 147) were recruited from the outpatients attending the Internal Medicine Department, community or schools through advertisement, and routine medical check-up. All individuals were screened for MetS. We used the United States National Cholesterol Education Program (NCEP) Expert Panel Adult Treatment Panel (ATP) III criteria as the recruitment criteria^22,23^. Individuals meet 3 or more of the following conditions will be diagnosed as MetS: 1) central obesity (waist circumference; WC ≥ 90 or 80 cm for Asian male and female, respectively); 2) triglycerides (TG) ≥ 1.7 mmol/L; 3) low level of high-density lipoprotein-cholesterol (HDL-C; ≤40 mg/dL for male and ≤50 mg/dL for female); 4) blood pressure (BP) ≥130/85 mmHg or receiving antihypertensive medication; 5) fasting blood glucose (FBG) ≥5.6 mmol/L or known history of T2DM. Individuals with symptomatic heart failure, liver cirrhosis, hepatic and renal failure, long-term steroid use, cancer, active infection, or other medical problems that would confound the results of this investigation were excluded. All control subjects were selected by FBG < 5.6 mmol/L and 2-h OGTT glucose < 7.8 mmol/L, no family history of T2DM, no any other major illnesses, and no medication that could have affected laboratory test results. Normal subjects were in good health and had normal kidney and liver function. This study was conducted in accordance with the Declaration of Helsinki, and was approved by the human research ethics committee of Chongqing Medical University, and informed consent was obtained from all subjects.

### Anthropometric and biochemical measurements

After 10–12 hours fasting overnight, all participants were scheduled at the same time (0800–0900 h) and received the comprehensive physical examination. Height, body weight, WC, hip circumstance and BP were measured by the same observer, and the waist-to-hip ratio (WHR) and Body mass index (BMI, weight divided by height squared) were calculated. The percentage of body fat (FAT %) was measured by bioelectrical impedance (BIA-101; RJL Systems, Shenzhen, China). The homeostasis model assessment of IR (HOMA-IR) was calculated using the following equations: HOMA-IR = fasting insulin (mU/L) × FBG (mmol/L)/22.5^24^. IR was defined as HOMA-IR value ≥ 3.8^25^. A 75-g OGTT was performed between 0730 and 0830 h. Blood samples were drawn at 0, 30, 60, and 120 min for the demonstration of glucose, insulin, blood fat and BMP-9. Blood glucose and HbA1c were measured by the glucose-oxidase method and anion- exchange HPLC, respectively. Plasma insulin concentrations were measured in 317 individuals using chemiluminescence. Free fatty acids (FFAs) were measured with a commercial kit (Randox Laboratories Ltd., Antrim, UK). Total cholesterol (TC), high-density lipoprotein cholesterol (HDL-C), low-density lipoprotein cholesterol (LDL-C), and triglyceride (TG) were determined enzymatically using an autoanalyzer (Hitachi 747; Hitachi, Tokyo, Japan).

### Measurements of circulating BMP-9 levels

Plasma BMP-9 concentrations were measured in duplicate with an ELISA kit according to the manufacturer’s protocol (R&D Systems, Inc., Catalog number DY3209, MN, USA). Briefly, 100 µl plasma was applied to the test BMP-9 concentrations. Then, 100 µl of specific biotin-conjugated anti-human BMP-9 was added to each well and incubated at 25 °C for 2 h. Each well was then washed three times. Colorimetric reaction was performed for 20 min with the use of horseradish peroxidase–conjugated streptavidin as substrate. A calibration curve was constructed by plotting the absorbance values at 450 nm versus the BMP-9 concentrations of the calibrators, and concentrations of samples were determined using this calibration curve. The minimum detectable concentration of human BMP-9 is <15.6 µg/L. The intra- and inter-assay coefficients of variation (CV) were low than 5% and 10%, respectively. The linear range of the assay was 15.60–1,000 µg/L. As in previous study^10^, we did not find that serum lipids or protein have effect on BMP-9 detection.

### Statistical analysis

Data are expressed as means ± standard deviation (SD) or medium (25th and 75th percentiles). Kolmogorov-Smirnov test was applied to test the normality of distribution. Data that were not normally distributed were log transformed. The differences of the anthropometric measurements and other parameters between groups were tested by independent student *t*-test or ANOVA. spearman correlation coefficients, partial correlation coefficients and multiple linear regressions were calculated to analysis the relationship between BMP9 and other parameters. Binary logistic regression model was applied to control the possible confounding variables and assess the relationship between BMP9 levels and MetS. Odds ratio (OR) and 95% confidence interval (CI) were also calculated. Logistic regression analysis was also used to assess and compare the odds of developing MetS of different BMP-9 tertile categories. The trends of BMP-9 levels associated with MetS were analyzed using the Cochran-Armitage trend test and the row mean score difference. Receiver operating characteristics (ROC) curves of BMP-9 levels were constructed to determine the optimal cutoff point for the prediction of MetS and IR. *P* value < 0.05 considered statistically significant.

## References

[1] Han TS, Lean MEJ (2016). A clinical perspective of obesity, metabolic syndrome and cardiovascular disease. JRSM Cardiovasc Dis..

[2] Sattar N (2003). Metabolic syndrome with and without C-reactive protein as a predictor of coronary heart disease and diabetes in the West of Scotland Coronary Prevention Study. Circulation..

[3] Girman CJ (2004). The metabolic syndrome and risk of major coronary events in the Scandinavian Simvastatin Survival Study (4S) and the Air Force/Texas Coronary Atherosclerosis Prevention Study (AFCAPS/TexCAPS). Am. J. Cardiol..

[4] Esposito K, Chiodini P, Colao A, Lenzi A, Giugliano D (2012). Metabolic syndrome and risk of cancer: a systematic review and meta-analysis. Diabetes Care..

[5] Pothiwala P, Jain SK, Yaturu S (2009). Metabolic syndrome and cancer. Metab Syndr Relat Disorders..

[6] Guallar-Castillon P (2014). Magnitude and management of metabolic syndrome in Spain in 2008–2010: the ENRICA study. Rev Esp Cardiol..

[7] Prasad DS, Kabir Z, Dash AK, Das BC (2012). Prevalence and risk factors for metabolic syndrome in Asian Indians: a community study from urban eastern India. J Cardiovasc Dis Res..

[8] Ford ES, Li C, Zhao G, Pearson WS, Mokdad AH (2008). Prevalence of the metabolic syndrome among U.S. adolescents using the definition from the International Diabetes Federation. Diabetes Care..

[9] Eckel RH, Alberti KG, Grundy SM, Zimmet PZ (2010). The metabolic syndrome. Lancet..

[10] Bidart M (2012). BMP9 is produced by hepatocytes and circulates mainly in an active mature form complexed to its prodomain. Cell Mol Life Sci..

[11] Miller AF, Harvey SA, Thies RS, Olson MS (2000). Bone morphogenetic protein-9. An autocrine/ paracrine cytokine in the liver. J Biol Chem..

[12] Breitkopf-Heinlein K (2017). BMP-9 interferes with liver regeneration and promotes liver fibrosis. Gut..

[13] Sharff KA (2009). Hey1 basic helix-loop-helix protein plays an important role in mediating BMP9-induced osteogenic differentiation of mesenchymal progenitor cells. J Biol Chem..

[14] David L, Feige JJ, Bailly S (2009). Emerging role of bone morphogenetic proteins in angiogenesis. Cytokine Growth Factor Rev..

[15] Lopez-Coviella I, Berse B, Krauss R, Thies RS, Blusztajn JK (2000). Induction and maintenance of the neuronal cholinergic phenotype in the central nervous system by BMP-9. Science..

[16] Song JJ (1995). Bone morphogenetic protein-9 binds to liver cells and stimulates proliferation. Endocrinology..

[17] Chen C (2003). An integrated functional genomics screening program reveals a role for BMP-9 in glucose homeostasis. Nat Biotechnol..

[18] Kuo MM (2014). BMP-9 as a potent brown adipogenic inducer with anti- obesity capacity. Biomaterials..

[19] Luo Y (2017). Decreased circulating BMP-9 levels in patients with Type 2 diabetes is a signature of insulin resistance. Clin Sci (Lond)..

[20] Caperuto LC (2008). Modulation of bone morphogenetic protein-9 expression and processing by insulin, glucose, and glucocorticoids: possible candidate for hepatic insulin-sensitizing substance. Endocrinology..

[21] Papandreou D (2009). Is there any association between high-density lipoprotein, insulin resistance and non-alcoholic fatty liver disease in obese children?. Int J Food Sci Nutr..

[22] Cleeman JI (2001). Executive summary of the third report of the National Cholesterol Education Program (NCEP) expert panel on detection, evaluation, and treatment of high blood cholesterol in adults (Adult Treatment Panel III). JAMA..

[23] Alberti KG (2009). Harmonizing the metabolic syndrome: a joint interim statement of the international diabetes federation task force on epidemiology and prevention; national heart, lung, and blood institute; American heart association; world heart federation; international atherosclerosis society; and international association for the study of obesity. Circulation..

[24] Report of the expert committee on the diagnosis and classification of diabetes mellitus. *Diabetes Care*. **20**, 1183–1197 (1997). 10.2337/diacare.20.7.1183 9203460

[25] Matthews DR (1985). Homeostasis model assessment: insulin resistance and beta-cell function from fasting plasma glucose and insulin concentrations in man. Diabetologia..

